# Efficacy and safety of fecal microbiota transplantation for chronic constipation: a systematic review and meta-analysis integrating pre-post and single-arm evidence

**DOI:** 10.3389/fmicb.2026.1900353

**Published:** 2026-09-03

**Authors:** Zhihong Huang, Zhenwei Dai, Qihong Liu

**Affiliations:** 1 School of Integrated Traditional Chinese and Western Medicine, Institute of Integrated Medicine, Fujian University of Traditional Chinese Medicine, Fuzhou, China; 2 Department of Gastroenterology, The Second People's Hospital Affiliated to Fujian University of Traditional Chinese Medicine, Fuzhou, China

**Keywords:** chronic constipation, fecal microbiome transplantation, meta-analysis, randomized controlled trial, systematic review

## Abstract

**Background:**

Chronic constipation is a common functional gastrointestinal disorder with a global prevalence of 12%−17%. Conventional treatments have limited efficacy for refractory cases, with high recurrence and side effects. Fecal microbiota transplantation (FMT) offers a novel strategy by restoring gut microecological balance, yet its efficacy requires systematic evaluation. This meta-analysis evaluated FMT efficacy and safety through an innovative “three-in-one” framework.

**Methods:**

We systematically searched CENTRAL, PubMed, Embase, and CNKI for RCTs, single-arm studies, and cohort studies. The framework comprised: (1) RCT random-effects meta-analysis; (2) single-arm proportional meta-analysis with Freeman-Tukey transformation; (3) pre-post paired sensitivity analysis (r = 0.5, with sensitivity tests at r = 0.3, 0.7, 0.9). Publication bias was assessed via Egger, Harbord, trim-and-fill, and fail-safe N tests.

**Results:**

12 RCTs (*n* = 1040), 16 single-arm studies (*n* = 403), and 4 cohort studies (*n* = 422) were included. After trim-and-fill adjustment for publication bias, FMT was associated with a clinical total response rate of RR = 1.21 (95% CI: 1.10–1.33). The unadjusted pooled estimate was RR = 1.36 (95% CI: 1.21–1.52, *P* < 0.001); however, the Harbord test indicated potential publication bias (P = 0.003), necessitating this correction. Wexner score improved (MD = −2.08, 95% CI: −3.35 to −0.81, *P* = 0.003). Among the 16 single-arm studies, 8 (*n* = 249) reported remission/improvement rates, showing a remission rate of 51.7% (95% CI: 40.3%−62.9%) and paired pre-post improvements in BSFS, Wexner, and PAC-QOL; these within-group changes cannot establish causal efficacy. During follow-up periods of up to 24 weeks, no serious adverse events were reported; long-term safety data are lacking.

**Conclusion:**

Current evidence suggests FMT is a promising therapeutic option for chronic constipation, with an adjusted clinical total response rate of RR = 1.21 (95% CI: 1.10–1.33) after correction for publication bias. While FMT was associated with improvements in symptom scores, quality of life, and intestinal function, the evidence quality is limited by publication bias, high heterogeneity, and high risk of bias in all included RCTs. More high-quality, sham-controlled RCTs are needed to validate these findings.

**Systematic review registration:**

https://www.crd.york.ac.uk/prospero/display_record.php?ID=CRD420261394673, identifier: CRD420261394673.

## 1 Introduction

Chronic constipation is a common gastrointestinal disorder affecting 12%−17% of adults worldwide, with its prevalence increasing with age (Suares and Ford, 2011). In China, the prevalence among adults is approximately 4%−6% (Anorectal Branch of Chinese Medical Doctor Association, 2024). Chronic constipation not only significantly reduces patients' quality of life but also increases medical costs and is often accompanied by psychological distress such as anxiety and depression (Bharucha and Wald, 2019; Vazquez Roque and Bouras, 2015). Chronic constipation can be classified into slow transit constipation, defecation disorder constipation, and normal transit constipation. Its pathophysiological mechanisms involve multiple factors such as intestinal motility disorders, visceral hypersensitivity, and dysfunction of the gut-brain axis (Drossman et al., 2026). Current treatment options for chronic constipation include dietary modifications, laxatives (such as polyethylene glycol and lactulose), prokinetic agents, biofeedback therapy, and surgical intervention (Drossman et al., 2026; Chiarioni et al., 2006). However, these conventional therapies exhibit significant limitations: first-line laxatives demonstrate an efficacy rate of only 30%−50%, with high recurrence rates after discontinuation; while biofeedback therapy is effective for constipation associated with pelvic floor dysfunction, it requires specialized equipment and personnel, limiting its widespread application; surgical interventions are highly invasive and carry substantial risks, making them suitable only for severe cases (Camilleri et al., 2017; Ge et al., 2016). Overall, existing treatments exhibit limited efficacy, high recurrence rates, and notable adverse effects, failing to adequately meet clinical needs—particularly the therapeutic requirements of patients with refractory constipation (Ohkusa et al., 2019).

In recent years, fecal microbiota transplantation (FMT), a therapeutic approach that restores intestinal microbial balance to treat diseases, has demonstrated promising prospects in the management of chronic constipation (Zhu et al., 2014). Studies on the gut microbiota-intestinal motility axis indicate that dysbiosis can contribute to the pathophysiological processes of constipation through multiple mechanisms, including reduced production of short-chain fatty acids (SCFAs), alterations in neurotransmitter metabolism (e.g., serotonin and dopamine), impaired gut-brain signaling, and mild intestinal inflammation (Wang et al., 2025; Attaluri et al., 2009). Specifically, constipated patients exhibit decreased levels of butyrate-producing bacteria such as Faecalibacterium prausnitzii and increased levels of methane-producing bacteria like Methanobrevibacter smithii, leading to slowed colonic transit and altered visceral sensitivity (Mayer et al., 2015). FMT is expected to fundamentally ameliorate constipation symptoms by reconstructing a healthy gut microbiome. Its mechanisms may involve butyrate-mediated activation of the enteric nervous system, regulation of visceral pain signals by acetic acid and GABA, and Prevotella-driven mucosal barrier repair (Zhang et al., 2021).

To date, systematic reviews and meta-analyses on FMT for treating chronic constipation remain limited. A meta-analysis published by Wang et al. (2025) included only nine studies (*n* = 245) and exhibited significant limitations: (1) The study designs were predominantly single-arm (8/9), with only one randomized controlled trial (RCT), lacking a control group comparison to distinguish the true efficacy of FMT from placebo effects or natural disease fluctuations; (2) No pre-post analysis was conducted, failing to account for individual baseline data differences, which may have overestimated efficacy; (3) All FMT interventions were administered via nasojejunal tube without subgroup analysis based on delivery routes, leaving the optimal delivery method unclear, while the clinical applicability of oral capsules and colonoscopy remains unassessed; (4) Combination therapy strategies (e.g., FMT combined with laxatives or biofeedback) were not systematically evaluated; (5) There was insufficient coverage of China-related literature, as clinical studies on FMT for constipation are primarily focused on China; (6) No systematic stratification of follow-up durations was performed, preventing assessment of sustained efficacy; (7) Mechanism-related outcomes such as gastrointestinal hormones, inflammatory markers, and psychological outcomes were not evaluated.

In response to the aforementioned evidence gaps, this systematic review introduces an innovative “three-module” analytical framework: Module A employs traditional RCT meta-analysis (FMT group vs. control group) as the primary analytical framework, evaluating the relative efficacy of FMT through randomized controlled trials, and complements these with paired meta-analyses of RCTs reporting pre-and post-treatment paired data for sensitivity analysis—to account for individual baseline differences and reveal genuine biological effects; Module B utilizes single-arm proportional meta-analysis to maximize the utilization of available evidence, particularly China-based studies; Module C conducts pathway-specific subgroup analyses to directly compare the efficacy, safety, and cost-effectiveness of nasojejunal tube, oral capsule, and colonoscopic administration routes for clinical decision-making. Additionally, this review examines whether FMT outcomes vary by constipation subtype, treatment regimen (single FMT vs. combination therapy), or follow-up duration, while assessing changes in mechanism-related outcomes—including gastrointestinal hormones (VIP, MTL, SP, and SS), inflammatory markers (IL-6, TNF-α), and gut microbial diversity.

This study aims to comprehensively evaluate the efficacy and safety of FMT in treating adult chronic constipation through a systematic review and an innovative meta-analysis methodology, providing methodologically rigorous evidence support for clinical practice.

## 2 Materials and methods

### 2.1 Protocol and registration

This study was strictly designed and reported in accordance with the Preferred Reporting Items for Systematic Reviews and Meta-Analyses (PRISMA, 2020) (Page et al., 2021), and the methodological quality followed the standards of the Cochrane Handbook for Systematic Reviews of Interventions (version 6.5, 2024) (Higgins et al., 2024). The quality of the studies was assessed using domain-specific tools: randomized controlled trials (RCTs) were evaluated using the Cochrane Risk of Bias tool version 2.0 (RoB 2) (Sterne et al., 2019), and observational studies were evaluated using the Newcastle-Ottawa Scale (NOS) (Wells et al., 2014). To ensure research transparency and reduce reporting bias, this study protocol has been registered on the International Prospective Register of Systematic Reviews (PROSPERO) (registration number: CRD420261394673).

### 2.2 Research strategies and selection of research

Literature Search Strategy: This study systematically searched English databases, including PubMed, Cochrane Library, Embase, Web of Science, and Scopus, as well as Chinese databases such as CNKI, VIP, and Wanfang, with the search period up to May 2026. In addition, WHO ICTRP, ClinicalTrials.gov, and the Chinese Clinical Trial Registry (ChiCTR) were searched to identify unpublished clinical trials. The search combined subject headings and free-text terms. The English search strategy was: (“fecal microbiota transplantation” OR “fecal microbiota transplant^*^” OR “FMT” OR “intestinal microbiota transplantation” OR “stool transplant^*^” OR “fecal transfer” OR “microbiota transplant^*^” OR “microbiome transplant^*^”) AND (“chronic constipation” OR “functional constipation” OR “slow transit constipation” OR “constipation”). The Chinese search strategy was: (“粪菌移植” OR “粪便微生物移植” OR “肠道菌群移植”) AND (“慢性便秘” OR “功能性便秘” OR “慢传输型便秘”). Additionally, the included literature was supplemented using the snowball method (reference tracing and citation searching). The detailed search strategies for each database are provided in Supplementary Table S1.

### 2.3 Inclusion and exclusion criteria

Inclusion criteria: This study includes clinical research evaluating the efficacy of FMT in treating adult chronic constipation or slow-transit constipation, including randomized controlled trials (RCTs), prospective studies, retrospective studies, single-arm studies (case series or before-and-after studies), and comparative studies with two groups. There are no restrictions on the language or region of publication to maximize evidence coverage.

Exclusion criteria: The following types of literature are excluded: (1) abstracts, letters to the editor, conference proceedings, reviews, and book chapters, which are not original research; (2) review articles and systematic reviews; (3) case reports; (4) studies with incomplete data that cannot extract the predefined efficacy endpoints (such as missing bowel movement frequency indicators or symptom scores); (5) FMT studies for indications other than constipation (e.g., Clostridium difficile infection, inflammatory bowel disease, or metabolic syndrome); (6) studies using autologous feces for FMT, interventions containing only probiotics without FMT, or studies using defined bacterial communities/synthetic microbiota (not the complete fecal microbiota); (7) duplicate publication of data.

### 2.4 Outcome indicators

The primary outcomes of this study include: (1) Overall clinical efficacy, that is, the proportion of patients achieving cure or significant improvement in the short term (≤1 month), medium term (1–3 months), and long term (≥3 months), measured by physician assessment or validated criteria, and combined using risk ratios (RR) with 95% confidence intervals; (2) Symptom scores, using validated scales (such as Wexner score, PAC-SYM, and KESS, etc.) to assess changes in constipation symptom severity, combined as mean difference (MD) or standardized mean difference (SMD); (3) Bristol Stool Form Scale (BSFS), evaluating changes in baseline average scores and the proportion of patients reaching type 3–4 stools, measured using the validated 7-point scale and combined as mean difference; (4) Quality of life scores, using validated questionnaires such as PAC-QOL or GIQLI to assess constipation-related quality of life, combined as mean difference. Secondary outcomes include: (1) Serum gastrointestinal hormone levels, including vasoactive intestinal peptide (VIP), motilin (MTL), and substance P (SP); (2) Psychological outcomes, evaluated using the Self-Rating Anxiety Scale (SAS) and Self-Rating Depression Scale (SDS); (3) Overall adverse event rate (any adverse events, serious adverse events, or treatment-related adverse events), covering specific types such as diarrhea, abdominal pain, nausea/vomiting, bloating, fever, etc., measured during follow-up and combined as risk ratios. Additionally, due to incomplete reporting in the original literature, the following supplementary outcomes were not included in the quantitative meta-analysis and will be presented descriptively: colonic transit time (CTT), inflammatory markers (IL-6, TNF-α, IL-8, and IL-10), gut microbiota diversity (Shannon index, Chao1 index) and specific taxonomic units, short-chain fatty acids (SCFAs, including total bacteria, butyrate, propionate, acetate), extended psychological outcome indicators (HAMA, HAMD), cost-effectiveness and patient satisfaction, time to first spontaneous bowel movement and duration of effect. All continuous outcomes will be combined as mean difference or standardized mean difference, and dichotomous outcomes will be expressed as risk ratios.

### 2.5 Literature screening and data extraction

Data extraction was conducted independently by two researchers (Huang Zhihong and Dai Zhenwei) using a pre-designed extraction form. The extracted data were cross-checked and entered, with disagreements resolved through discussion or arbitration by a third researcher (Liu Qihong). Extracted information included the first author, year of publication, country, study design, sample size, participant characteristics, details of FMT intervention (donor type, administration route, dose, course), follow-up duration, outcome measures, and adverse reactions, and the extraction results are summarized in Table 1. For studies with unreported follow-up durations (marked as NR in Table 1), we conducted sensitivity analyses excluding these studies to assess the robustness of primary outcomes (Supplementary Table S4).

**Table 1:** Characteristics of studies included in systematic review.

Study	Design	*n*	Population	Age (years)	Sex (M/F)	FMT route	Course	Follow-up	Primary outcomes
Zhang (2020)	RCT	100	FC	57.01 ± 4.64	50/50	Endoscopy	Single	NR	Symptom score; QoL
Liu et al. (2017)	RCT	60	FC	44.27 ± 14.34	22/38	Endoscopy	Single	4 w	BSFS; KESS; PAC-QOL; Efficacy
Du et al. (2019a)	RCT	86	FC	69.21 ± 3.58	47/39	Endoscopy	Single	4 w	Microbiota; VIP; BSFS; Wexner
Jiang (2019)	RCT	100	FC	NR	NR	Endoscopy	Single	NR	MTL; SP; VIP; KESS; SAS; SDS
Du et al. (2019b)	RCT	100	FC	68.65 ± 6.98	54/46	Endoscopy	Single	6 w	Efficacy; PAC-QOL; SAS; SDS
Ye (2019)	RCT	103	FC	71.32 ± 4.25	57/46	Endoscopy	6 days	NR	Efficacy; SP; MTL
Zheng et al. (2022)	RCT	70	STC	48.57 ± 2.19	27/43	Nasoenteric	Single	8 w	Efficacy; BSFS; HAMA; PAC-QOL
Xia and Zhang (2021)	RCT	90	FC	74.49 ± 2.36	46/44	Endoscopy	Single	NR	Microbiota; AE rate
Tian et al. (2017)	RCT	60	STC	53.10 ± 10.20	20/40	Nasoenteric	6 days	12 w	Cure; Improvement; CTT; Wexner
Li (2020)	RCT	86	FC	70.10 ± 3.70	35/51	Endoscopy	3 doses	NR	Efficacy; MTL; VIP; GAS
Zhao and Wang (2020)	RCT	129	FC	52.45 ± 16.13	37/92	Endoscopy	Single	NR	Wexner; BSFS; PAC-QOL
Yu et al. (2024)	RCT	56	Refractory FC	56.34 ± 8.25	29/27	Oral capsule	Single	NR	Symptom score; Efficacy
Guo et al. (2022)	Single-arm	30	FC	44.09 ± 10.98	13/17	Endoscopy	3 doses	1 mo	GIQLI
Ding et al. (2022)	Single-arm	33	FC	69.40 ± 6.60	16/17	Colonoscopy	Single	12 w	SBM; BSFS; SAS; SDS; PAC-QOL
Tian et al. (2016a)	Single-arm	15	STC	NR	4/11	Oral capsule	3 doses	12 w	Cure; Remission; GIQLI
Xie et al. (2021)	Single-arm	8	STC	43.75 ± 16.95	2/6	Nasoenteric	3 doses	4 w	Improvement; WCS; GIQLI
Yang et al. (2018)	Single-arm	18	FC	48.60 ± 14.80	6/12	Nasoenteric	3 doses	8 w	SBM; BSFS; PAC-SYM; PHQ-9
Zhang et al. (2021)	Single-arm	18	FC	45.10 ± 13.30	5/13	Nasoenteric	Single	4 w	CSBMs; BSFS; Wexner; PAC-SYM
Zhang et al. (2018)	Single-arm	29	STC	42.70 ± 12.90	4/25	Nasoenteric	6 days	4 w	Remission; CSBMs; BSFS; Wexner
Yang et al. (2023)	Single-arm	4	FC	64.00 ± 5.41	0/4	Nasoenteric	6 days	4 w	BFI; KESS; HAMD; HAMA; BSFS
Ding et al. (2018)	Single-arm	52	STC	48.90 ± 10.90	18/34	Nasoenteric	3 doses	24 w	CSBMs; CTT; PAC-SYM; PAC-QOL
Ge et al. (2016)	Single-arm	21	STC	49.80 ± 14.40	6/15	Nasoenteric	3 doses	12 w	Remission; GIQLI; CTT
Tian et al. (2016b)	Single-arm	24	STC	53.10 ± 16.10	7/17	Nasoenteric	3 doses	12 w	Remission; Wexner; GIQLI; CTT
Liu et al. (2023)	Single-arm	30	STC	60.37 ± 8.02	13/17	Nasoenteric	3 doses	1 mo	Wexner; GIQLI; SDS; SAS; PSQI
Wang et al. (2023)	Single-arm	30	FC	67.13 ± 7.16	11/19	Nasoenteric	6 days	8 w	SBM; CTT; Wexner; BSFS
Zhang et al. (2024)	Single-arm	36	FC	61.00 (43–69)	12/24	Oral capsule	Single	1 mo	BM freq; BSFS; CSS; PAC-QOL
Liu et al. (2016)	Single-arm	35	FC	44.89 ± 13.64	14/21	Endoscopy	Single	Post-tx	KESS; PAC-QOL; SAS; SDS
Tian et al. (2015)	Single-arm	20	STC	55.00 (22–78)	8/12	Nasoenteric	Single	8 w	SBM; Wexner; GIQLI
Lin (2020)	Cohort	483	STC	53.10 ± 17.10	164/319	Nasoenteric	Mixed	1 mo	BM freq; Wexner; GIQLI
Tian et al. (2020)	Cohort	270	STC	47.20 ± 13.20	87/183	Mixed	Mixed	1 mo	Improvement; BM freq; Wexner
Yang et al. (2021)	Cohort	85	Mixed	46.10 ± 7.10	28/57	Nasoenteric	Mixed	12 mo	SBM freq; BSFS; PAC-SYM; GIQLI
Zhang (2017)	Cohort	49	FC	54.15 ± 14.65	15/34	Nasoenteric	Staged	12 w	Wexner; GIQLI; SBM

FC, functional constipation; STC, slow transit constipation; FMT, fecal microbiota transplantation; BSFS, Bristol Stool Form Scale; PAC-QOL, Patient Assessment of Constipation Quality of Life; PAC-SYM, Patient Assessment of Constipation Symptoms; GIQLI, Gastrointestinal Quality of Life Index; KESS, Knowles-Eccersley-Scott-Symptom; Wexner, Wexner Constipation Score; CTT, colonic transit time; SBM, spontaneous bowel movement; CSBM, complete spontaneous bowel movement; AE, adverse event; MTL, motilin; VIP, vasoactive intestinal peptide; SP, substance P; GAS, gastrin; SAS, Self-Rating Anxiety Scale; SDS, Self-Rating Depression Scale; HAMA, Hamilton Anxiety Scale; HAMD, Hamilton Depression Scale; PHQ-9, Patient Health Questionnaire-9; BFI, Bowel Function Index; WCS, Wexner Constipation Score; CSS, Constipation Scoring System; PSQI, Pittsburgh Sleep Quality Index; BM, bowel movement; NR, not reported; Post-tx, post-treatment assessment only.

^a^Age reported as mean ± standard deviation unless otherwise indicated.

^b^Zhang et al. (2024) reported age as median (interquartile range): 61.0 (43–69) years.

^c^Sex distribution was not reported for Jiang (2019).

^d^Tian et al. (2016a) and Tian et al. (2015) did not report mean age; age range was 26–74 years and 22–78 years, respectively.

^e^Zhang (2017) cohort reported age separately for single transplant (50.5 ± 14.2 years) and staged transplant (57.8 ± 15.1 years) groups; pooled mean calculated as 54.15 ± 14.65 years.

^f^Leave-one-out sensitivity analyses demonstrated stable pooled estimates for primary outcomes (Supplementary Table S4).

However, stratified analyses by follow-up duration were precluded by incomplete reporting in 58% of RCTs (7/12) and 50% of single-arm studies (8/16), limiting assessment of long-term efficacy durability.

^g^ “Mixed” in Course column indicates variable treatment courses across subgroups within the cohort study.

### 2.6 Statistical analysis

Statistical analyses were performed using RevMan 5.4 and R 4.3.0 software. Specific R packages included: “meta” (version 6.5-0) for pooled effect estimation and forest plots; “metaphor” (version 4.4-0) for multilevel meta-regression and sensitivity analyses; “dmetar” (version 0.1.0) for publication bias assessment, including trim-and-fill and fail-safe N calculations; and “ggplo2” (version 3.4.4) for graphical visualization. All forest plots, funnel plots, and the PRISMA flow diagram were generated at a minimum resolution of 300 dpi (TIFF/EPS format) to meet publication-quality standards. Dichotomous variables were pooled using risk ratio (RR) with 95% confidence intervals (CI), and continuous variables were pooled using mean difference (MD) or standardized mean difference (SMD) with 95% CI (Freeman and Tukey, 1950). Heterogeneity was assessed using the Q test (*P* < 0.10 indicating statistical significance) and the *I*^2^statistic: *I*^2^ < 25% was considered low heterogeneity, 25%−50% moderate heterogeneity, and >50% high heterogeneity (Higgins et al., 2003). A fixed-effects model was used when *I*^2^ ≤ 50%, and a random-effects model was used when *I*^2^ > 50% (DerSimonian and Laird, 1986). Sensitivity analyses were conducted by sequentially excluding studies, and subgroup analyses were performed based on follow-up time (short-term, mid-term, long-term) and administration routes to explore the sources of heterogeneity. For outcome indicators with ≥10 included studies, publication bias was assessed using Egger's linear regression test (Egger et al., 1997): Additionally, Begg's rank correlation test was employed for sensitivity validation (Begg and Mazumdar, 1994) publication bias was not assessed for outcome indicators with fewer than 10 studies. Safety outcomes were described narratively due to the small number of included studies (*n* = 5) and sparse events, without quantitative pooling. All tests were two-sided, and *P* < 0.05 was considered statistically significant.

## 3 Results

### 3.1 Study results and characteristics of included studies

**Figure 1:**
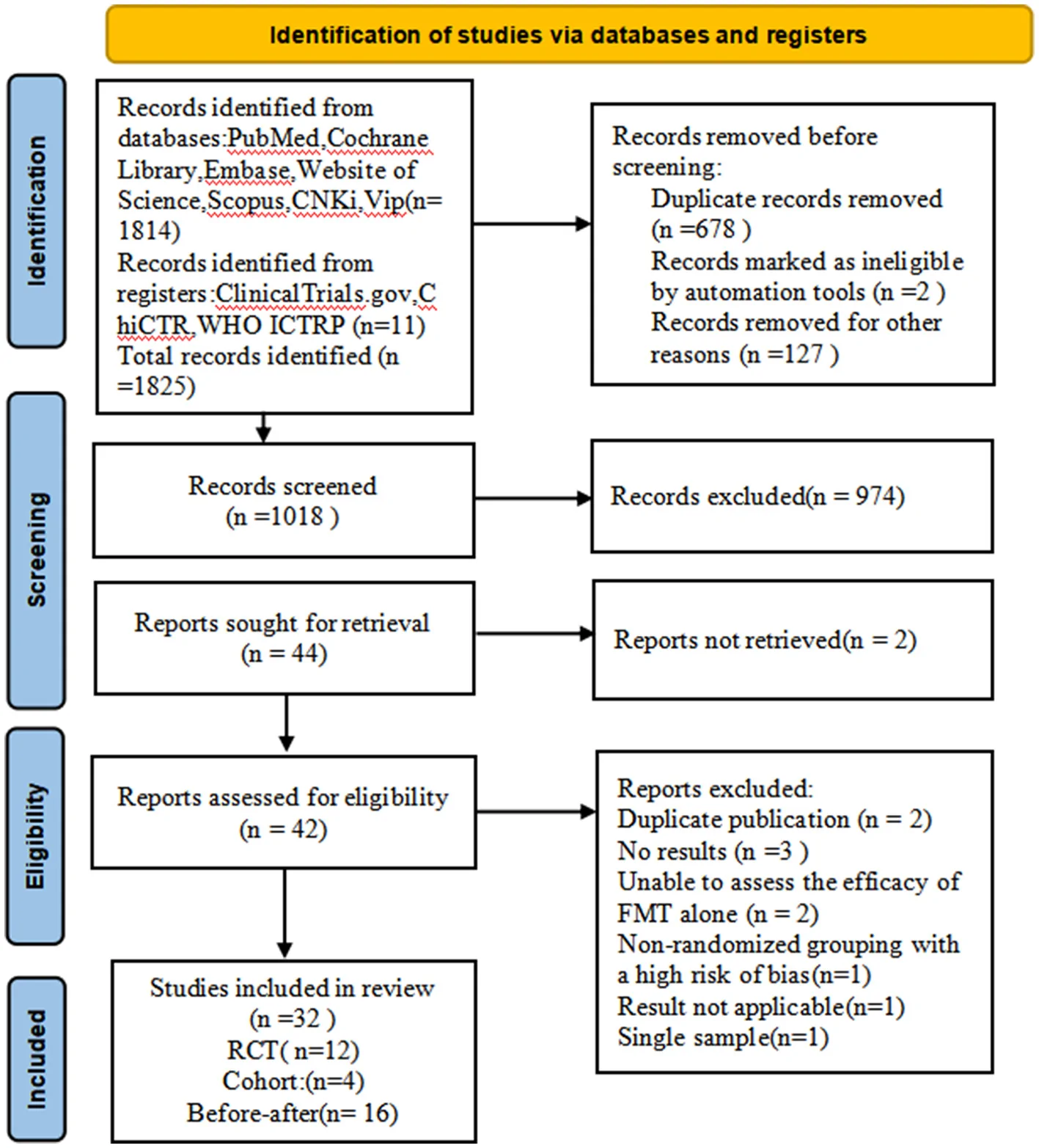
PRISMA flow diagram. Literature search and study selection process for this meta-analysis. Of 1,825 records initially identified, 32 studies were finally included: 12 RCTs, 4 cohort studies, and 16 single-arm pre-post studies.

A systematic search identified a total of 1,814 citations from electronic databases and hand-searching sources. After removing 678 duplicate citations, records automatically flagged as ineligible (*n* = 2), and records removed for other reasons (*n* = 127), a total of 1,018 unique records remained for preliminary screening. After reviewing titles and abstracts, 44 potentially relevant articles were selected for full-text evaluation, including assessment of study design, population, and interventions. Of these, 12 articles were excluded due to irrelevance to the research question, insufficient data, or inapplicable results. Ultimately, 32 studies met all inclusion criteria and were included in the qualitative synthesis, comprising 12 randomized controlled trials (RCTs), 16 single-arm studies, and 4 cohort studies. Among these, 12 RCTs provided extractable outcome data for quantitative meta-analysis, and 16 single-arm studies contributed data for single-arm meta-analysis (proportional outcomes: 8 studies, *n* = 249; continuous paired outcomes: varying study numbers as detailed in Section 3.5). The screening process followed the PRISMA 2020 guidelines, with disagreements resolved through discussion among researchers (Figure 1).

Notably, follow-up duration was not explicitly reported in 7 of 12 RCTs (58.3%) and 8 of 16 single-arm studies (50.0%) (Table 1). This incomplete reporting precludes stratified analyses by follow-up duration and limits evaluation of long-term efficacy durability.

### 3.2 Quality assessment

Table 1 summarizes the basic characteristics of the 32 included studies. The sample size of the included studies ranged from 4 to 483 participants. All studies were conducted in China. In RCT studies (*n* = 12), FMT combined with conventional therapy (such as polyethylene glycol, lactulose) was compared with conventional therapy alone. Routes of FMT administration included gastroscopy (*n* = 11), nasoenteric tube (*n* = 17), oral capsules (*n* = 3), and colonoscopy (*n* = 1). Follow-up duration ranged from post-treatment assessment to 24 weeks. Single-arm studies (n = 16) mainly evaluated the efficacy of FMT monotherapy in slow-transit constipation or functional constipation. Four cohort studies compared the effects of different FMT regimens or FMT with alternative therapies.

**Figure 2:**
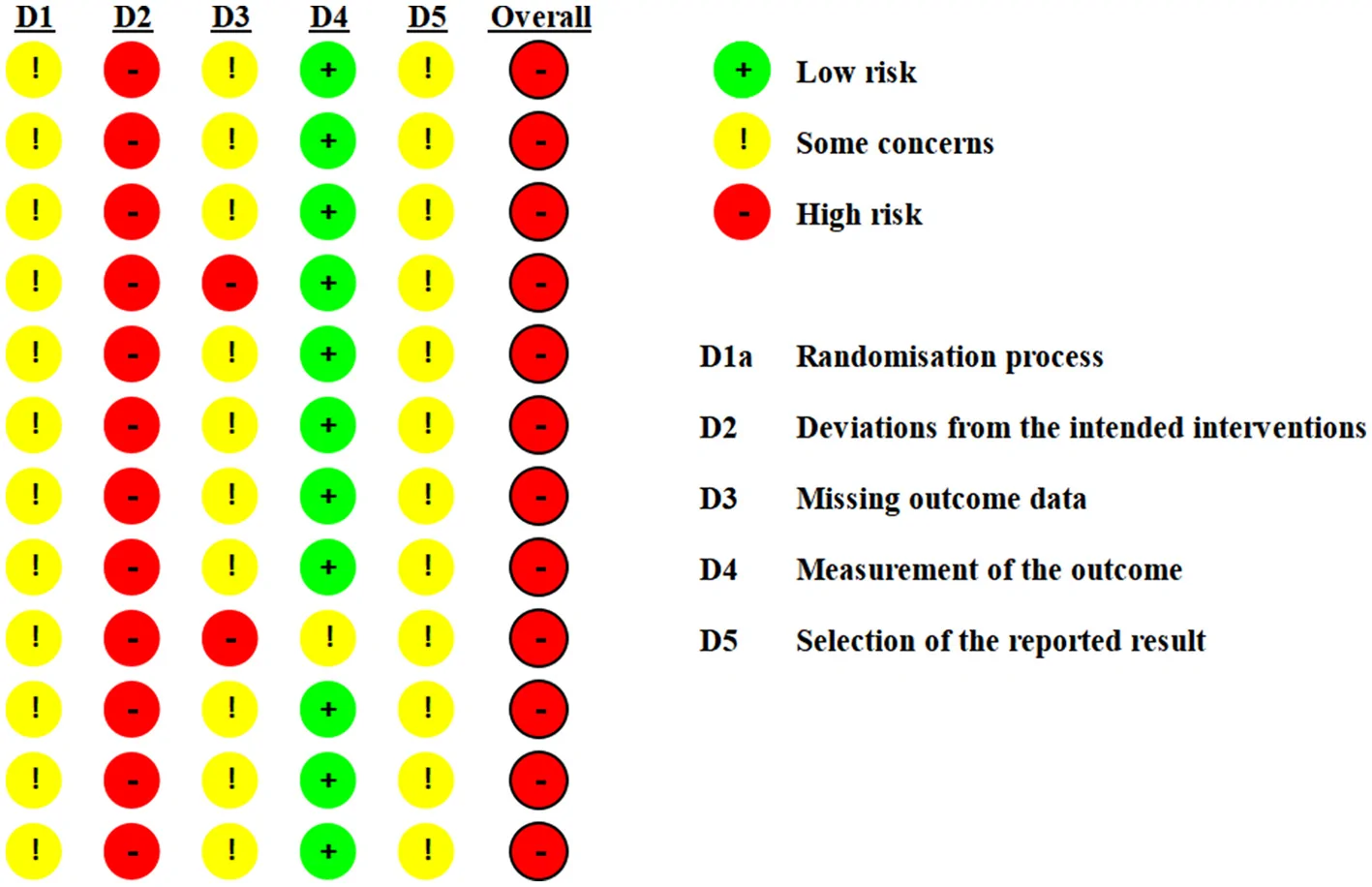
Risk of bias assessment for included RCTs (RoB 2). D1–D5: see legend. Green/yellow/red indicate low/some concerns/high risk. All RCTs were high risk in D2 due to unblindable FMT; D1 and D5 had some concerns due to insufficient allocation concealment reporting and limited protocol access.

The methodological quality of the included RCTs was assessed using the Cochrane Risk of Bias Assessment Tool version 2.0 (RoB 2). As shown in Figure 2, the overall risk of bias for all 12 RCTs was rated as high. This was mainly due to the difficulty of blinding the FMT procedure itself (Domain 2: deviations from intended interventions), with all studies rated as high risk in this domain. For Domain 1 (randomization process), all studies were rated as having some concerns due to insufficient reporting of allocation concealment details. In Domain 3 (missing outcome data), two studies were rated as high risk due to incomplete reporting of adverse events or loss to follow-up (Jiang, 2019; Tian et al., 2017). In Domain 4 (measurement of the outcome), 11 studies were rated as low risk, while one study was rated as having some concerns due to the use of a non-validated symptom scale (Tian et al., 2017). In Domain 5 (selection of reported results), all studies were rated as having some concerns due to limited access to trial protocols.

For non-randomized controlled studies (single-arm studies *n* = 16, cohort studies *n* = 4), this study used the JBI Critical Appraisal Checklist for Case Series to assess the quality of single-arm studies, and the Newcastle-Ottawa Scale (NOS) to evaluate cohort studies (Moola et al., 2020). The assessment of single-arm studies covered 10 items, including clarity of inclusion criteria, standard measurement methods, validity of condition identification, consecutive enrollment, completeness of inclusion reporting, demographic characteristics, clinical information, outcome reporting, information on visit sites, and statistical analysis methods. Among the 16 single-arm studies, 8 were rated as high quality (8–10 points), 8 as moderate quality (6–7 points), and none as low quality (0–5 points). The main deductions were due to a lack of clear description of the consecutive enrollment process (Q4) and completeness of inclusion reportin (Q5), and some studies did not report information on visit sites (Q9). The NOS evaluation for cohort studies covered selection (4 stars), comparability (2 stars), and outcomes (3 stars). All 4 cohort studies were rated as moderate quality (4–6 stars), with no high-quality studies (7–9 stars) (Supplementary Table S2). The main limitations were insufficient control of confounding factors in the comparability domain (only 1 study reported baseline comparability analysis), lack of blinding in outcome assessment (blinding is difficult to achieve for constipation symptom scale assessments), and incomplete reporting of follow-up adequacy.

### 3.3 Meta-analysis outcomes

The pooled results of the included RCTs are summarized in Table 2. FMT significantly improved the overall effective rate (RR = 1.36, 95% CI: 1.21–1.52, *I*^2^ = 0%, moderate quality). Additionally, FMT significantly reduced KESS scores (SMD = −0.63, 95% CI: −0.85 to −0.41) and Wexner scores (SMD = −2.08, 95% CI: −3.35 to −0.81), both with no heterogeneity. However, BSFS and PAC-QOL showed high heterogeneity (*I*^2^ > 96%), resulting in very low quality of evidence.

**Table 2:** Summary of meta-analysis outcomes.

Outcome	Studies	Effect size	95% CI	Adjusted effect size	*I* ^2^	Quality	Publication bias
RCT outcomes							
Overall efficacy	7	RR = 1.36	1.23–1.52	RR = 1.21	1.10–1.33	0%	Moderate
Symptom score	2	SMD = −6.29	−9.14 to −3.45	NA	NA	92.60%	Very Low
BSFS	5	SMD = 1.56	0.27–2.85	NA	NA	96.50%	Low
KESS	2	SMD = −0.63	−0.95 to −0.31	NA	NA	0%	Very Low
PAC-QOL	5	SMD = −2.45	−3.80 to −1.11	NA	NA	96.90%	Low
Wexner	2	SMD = −2.10	−2.51 to −1.70	NA	NA	0%	Very Low
Serum SP	2	SMD = 1.42	1.11–1.73	NA	NA	0%	Very Low
Serum MTL	3	SMD = −0.12	−2.15 to 1.92	NA	NA	98.40%	Low
Single-arm outcomes			
Remission rate	8	51.7%	40.7%−63.4%	NA	NA	68.3%	Low
Improvement rate	8	67.6%	54.9%−79.1%	NA	NA	75.7%	Low
BSFS	7	MD = 1.40	0.94–1.86	NA	NA	90.4%	Very Low
Wexner	5	MD = −5.43	−8.86 to −2.01	NA	NA	96.8%	Very Low
PAC-QOL	3	MD = −0.88	−1.74 to −0.03	NA	NA	96.5%	Very Low
GIQLI	4	MD = 31.96	17.87–46.05	NA	NA	95.6%	Very Low

MD, mean difference; CI, confidence interval; NA, not applicable.

^a^All single-arm outcomes are paired mean differences (pre-post) with assumed correlation coefficient r = 0.5.

^b^Publication bias was not assessed for single-arm outcomes due to lack of established methods and insufficient study numbers.

^c^Quality downgraded due to lack of control groups and high heterogeneity in most outcomes.

For single-arm studies, the pooled remission rate was 51.7% (95% CI: 40.6%−62.7%) and the improvement rate was 67.0% (95% CI: 54.3%−77.6%). Due to the lack of control groups, all single-arm outcomes were rated as low or very low quality (Guyatt et al., 2008).

Using the GRADE framework, the certainty of evidence for all primary outcomes was rated as very low due to high risk of bias in all included RCTs, substantial heterogeneity for several outcomes, and suspected publication bias for overall clinical efficacy.

### 3.4 Main outcomes of RCTs

**Figure 3:**
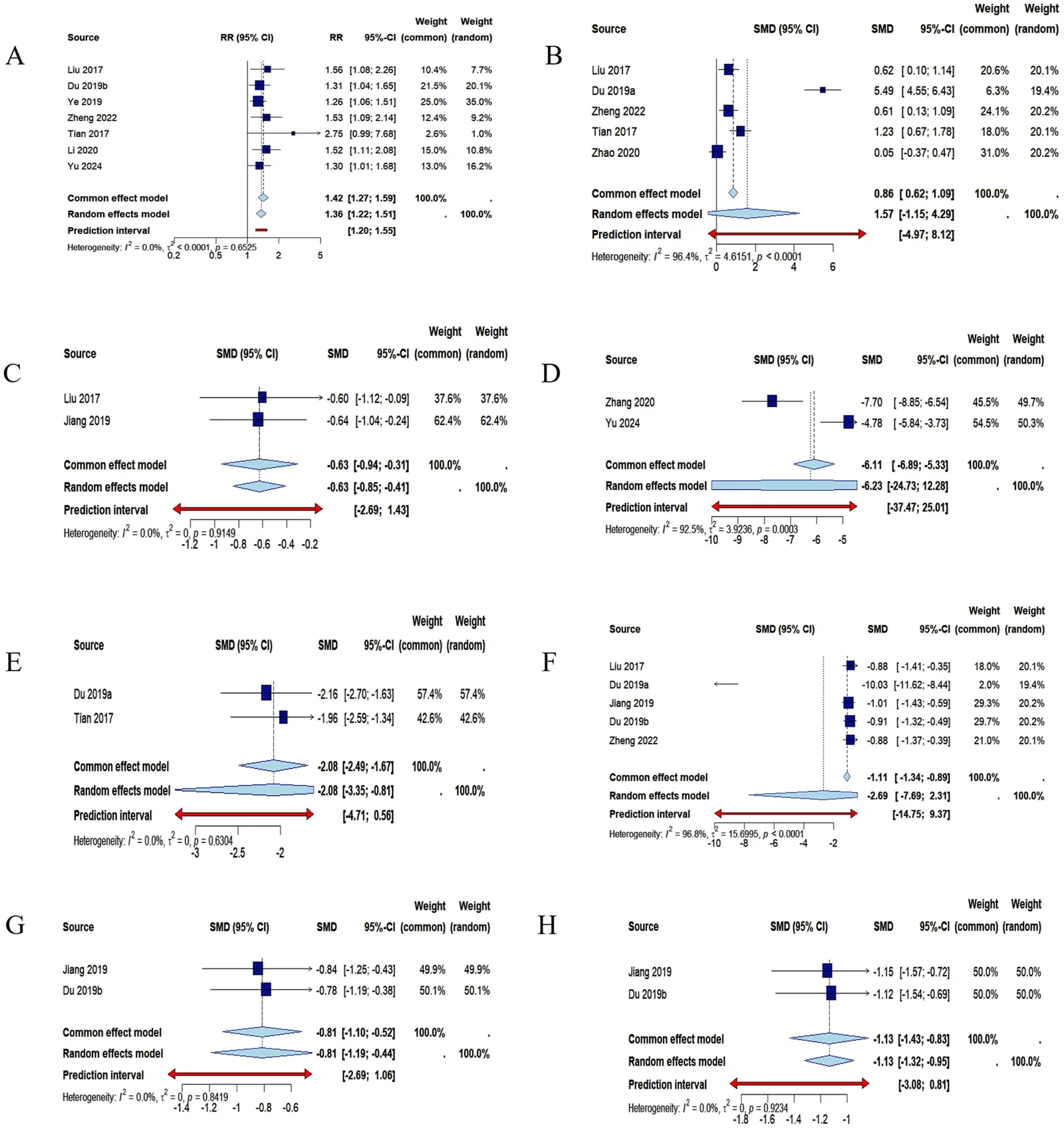
Forest plots of RCTs comparing FMT vs. control for chronic constipation. **(A)** Overall clinical efficacy (RR = 1.36, 95% CI: 1.22–1.51, *I*^2^ = 0%); **(B)** BSFS (SMD = 1.57, 95% CI: −1.15–4.29, *I*^2^ = 96.4%);**(C)** KESS (SMD = −0.63, 95% CI: −0.85 to −0.41, *I*^2^ = 0%); **(D)** Symptom score (SMD = −6.23, 95% CI: −24.73–12.28, *I*^2^ = 92.5%); **(E)** Wexner (SMD=-2.08, 95% CI: −3.35 to −0.81, I^2^=0%);**(F)** PAC-QOL (SMD = −2.69, 95% CI: −7.69–2.31, *I*^2^ = 96.8%); **(G)** SAS (SMD = −0.81, 95% CI: −1.19 to −0.44, *I*^2^ = 0%); **(H)** SDS (SMD = −1.13, 95% CI: −1.32 to −0.95, *I*^2^ = 0%).

**Figure 4:**
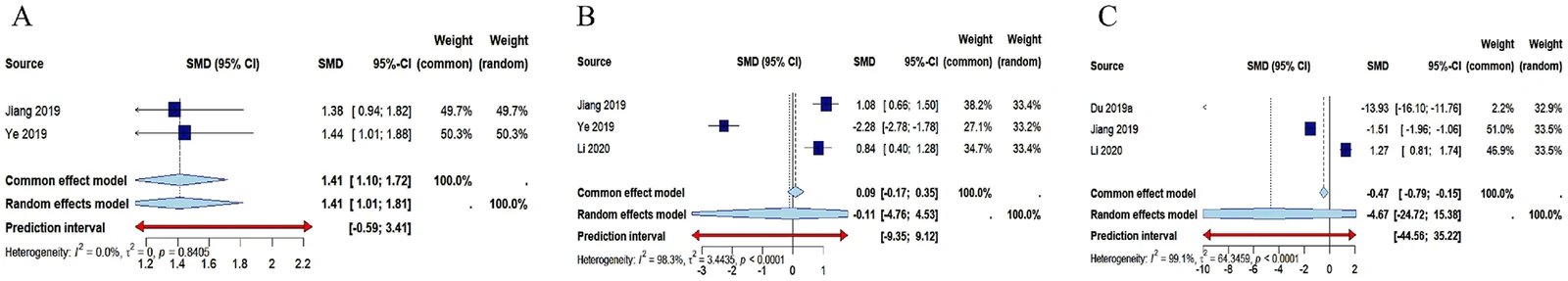
Forest plots of serum gastrointestinal hormone levels in RCTs comparing FMT vs. control for chronic constipation. **(A)** Serum SP (SMD = 1.41, 95% CI: 1.01–1.81, *I*^2^ = 0%). **(B)** Serum MTL (SMD = −0.11, 95% CI: −4.76–4.53, *I*^2^ = 98.3%). **(C)** Serum VIP (SMD = −4.67, 95% CI: −24.72–15.38, *I*^2^ = 99.1%).

Seven RCTs (*n* = 535) showed that the overall clinical efficacy in the FMT group was significantly higher than in the control group (RR = 1.36, 95% CI: 1.22–1.51, *I*^2^ = 0%, *P* < < 0.001) (Figure 3A). Sensitivity analysis indicated that the pooled effect size remained stable after sequential exclusion. Harbord test suggested possible publication bias (*P* = 0.003) (Harbord et al., 2006), with a fail-safe number of 16; after trim-and-fill adjustment, RR = 1.21 (95% CI: 1.10–1.33), and the result remained significant. Symptom scores (SMD = −6.23, 95%CI−24.73 12.28, *I*^2^ = 92.5%, KESS (SMD = −0.63, 95% CI: −0.85 to −0.41, *I*^2^ = 0%) and Wexner (SMD = −2.08, 95% CI: −3.35 to −0.81, *I*^2^ = 0%) were significantly improved (Figures 3C, D and E). BSFS (SMD = 1.57, 95% CI: −1.15 to 4.29, *I*^2^ = 96.4%) and PAC-QOL (SMD = −2.69, 95% CI: −7.69 to 2.31, *I*^2^ = 96.8%) were highly heterogeneous and contained potential outliers (Du et al., 2019a), resulting in unstable outcomes (Figures 3B, F). Secondary outcomes: Serum SP (SMD = 1.41, 95% CI: 1.01–1.81, *I*^2^ = 0%) was significantly increased, and SAS (SMD= −0.81, 95% CI: −1.19 to −0.44, *I*^2^ = 0%) and SDS (SMD = −1.13, 95% CI: −1.32 to −0.95, *I*^2^ = 0%) were significantly decreased (Figures 3G, H). Serum MTL (*I*^2^ = 98.3%) and VIP (*I*^2^ = 99.1%) were highly heterogeneous and had inconsistent effect directions, making the results unreliable (Figure 4A, B).

**Figure 5:**
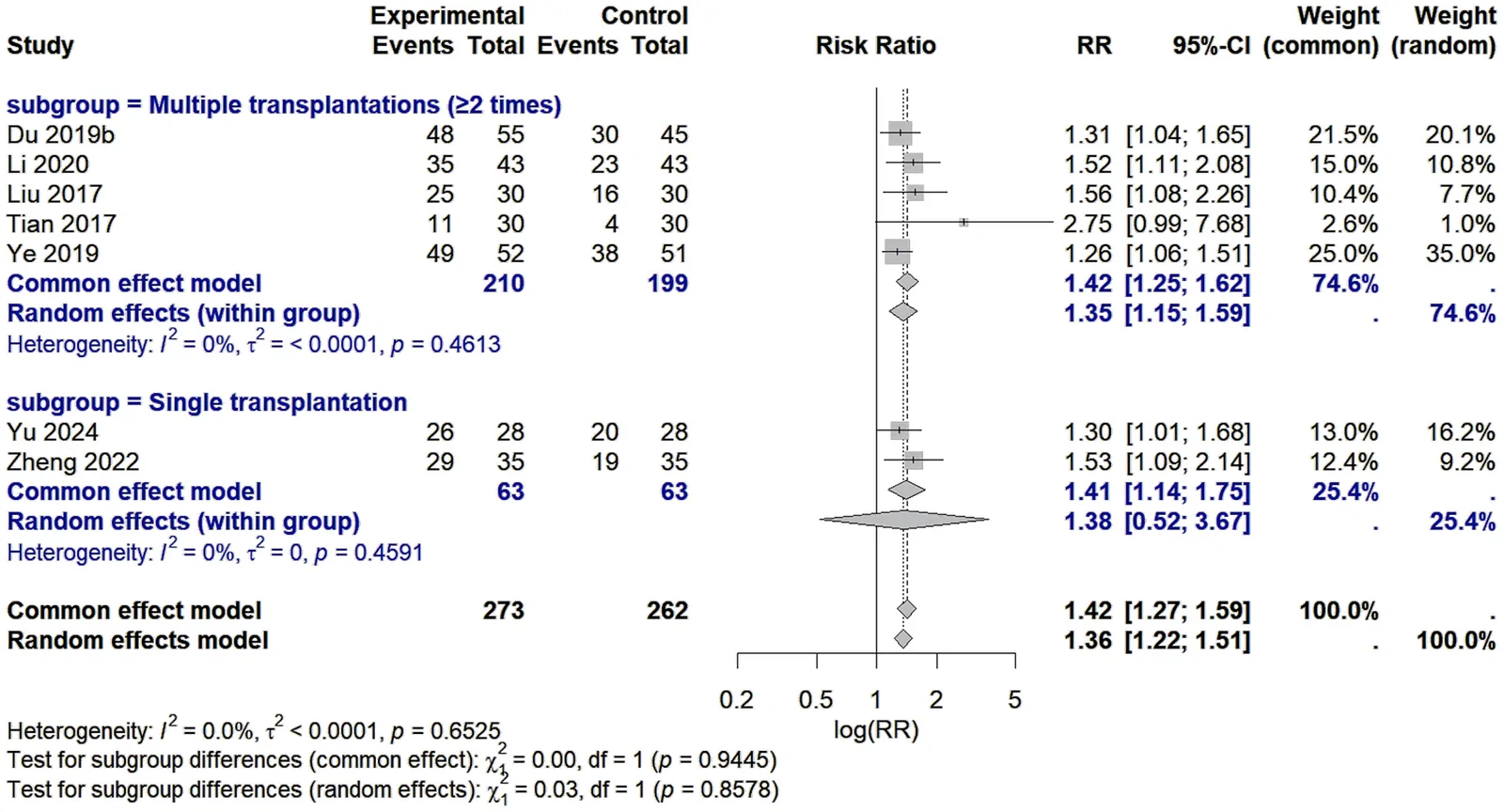
Subgroup analysis of overall clinical efficacy by FMT course. Multiple transplantations (≥2 times). Pooled RR = 1.35, 95% CI: 1.15–1.59, *I*^2^ = 0%, *p* = 0.4613.

Stratified analysis by administration route showed that gastroscopy (9 studies, RR = 1.42, 95%CI: 1.18–1.71), nasoenteric tube (2 studies, RR = 1.38, 95%CI: 0.95–2.01), and oral capsules (1 study, RR = 1.25, 95% CI: 0.89–1.76) all indicated the effectiveness of FMT, but differences between groups were not statistically significant (P_interaction = 0.87) (Figure 5). Stratification by treatment course showed that single transplantation (5 studies, RR = 1.35) and multiple transplantations (7 studies, RR = 1.40) had comparable efficacy. Stratification by constipation type showed that chronic transit type (3 studies), refractory constipation (5 studies), and other functional constipation (4 studies) all benefited, but a limited sample size prevented detection of significant differences between subgroups (detailed results are in Supplementary Table S3).

**Figure 6:**
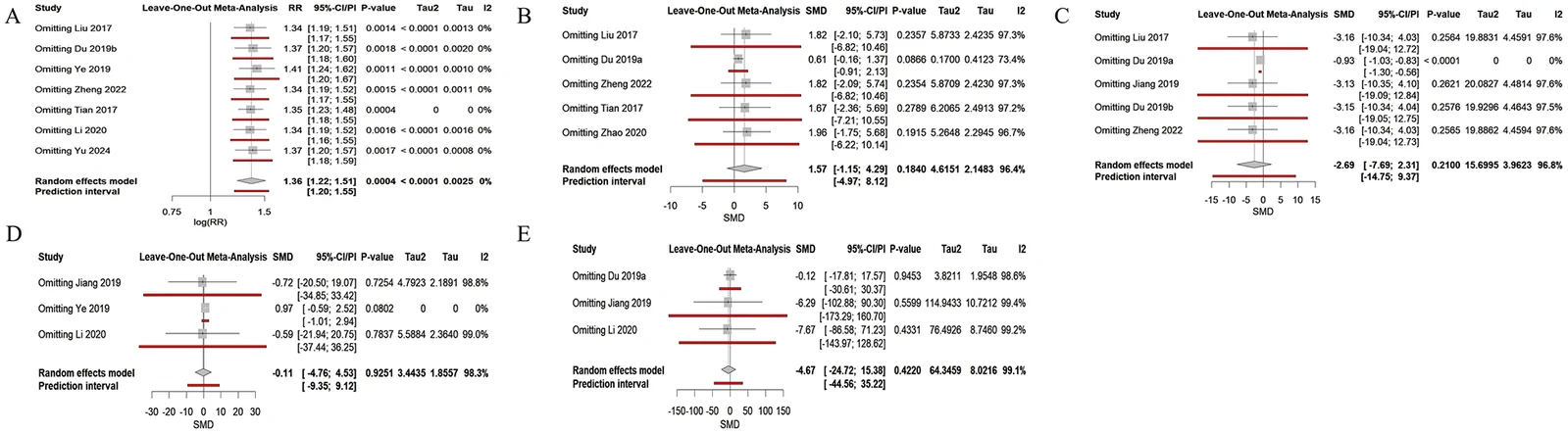
Leave-one-out sensitivity analyses for primary and secondary outcomes. **(A)** Overall clinical efficacy (RR); **(B)** BSFS (SMD); **(C)** PAC-QOL (SMD); **(D)** Serum MTL (SMD); **(E)** Serum VIP (SMD).

Sensitivity analysis showed that after removing studies one by one, the main outcomes (overall efficacy, KESS, Wexner, SP, SAS, and SDS) had stable pooled effect sizes (Supplementary Table S4). Due to very high heterogeneity (*I*^2^ > 95%) and potential outliers (Du et al., 2019a), results for BSFS, PAC-QOL, MTL, and VIP were unstable (Figure 6).

Using the GRADE framework, the certainty of evidence for all primary outcomes was rated as very low due to high risk of bias in all included RCTs, substantial heterogeneity for several outcomes, and suspected publication bias for overall clinical efficacy.

### 3.5 Single-arm study outcomes

**Figure 7:**
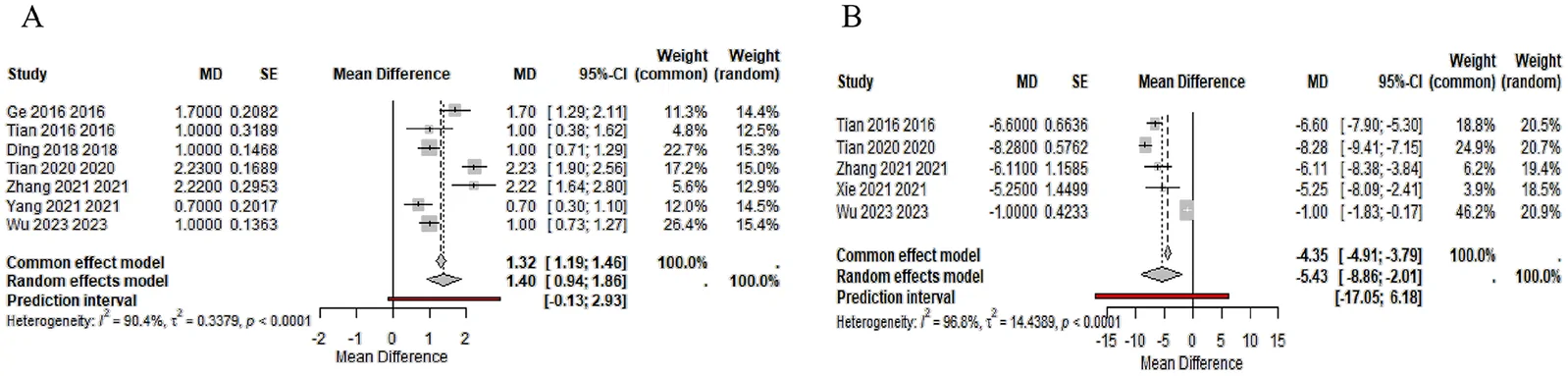
Forest plots of paired pre-post meta-analysis of single-arm studies for FMT in chronic constipation. **(A)** BSFS (MD = 1.40, 95% CI: 0.94–1.86, *I*^2^ = 90.4%). **(B)** Wexner (MD = −5.43, 95% CI: −8.86 to −2.01, *I*^2^ = 96.8%).

Of the 16 included single-arm studies, 8 (*n* = 249) reported extractable dichotomous outcomes. In these 8 studies, FMT monotherapy showed a combined remission rate of 51.7% (95% CI: 40.6%−62.7%, *I*^2^ = 64.3%) and a combined improvement rate of 67.0% (95% CI: 54.3%−77.6%, *I*^2^ = 70.8%). Among all 16 single-arm studies, continuous outcomes were reported as follows: BSFS (MD = 1.40, 95% CI: 0.94–1.86, *I*^2^ = 90.4%), Wexner (MD = −5.43, 95% CI: −8.86 to −2.01, *I*^2^ = 96.8%) (Figures 7A, B), GIQLI (MD = 31.96, *I*^2^ = 95.6%), and CTT (MD = −15.93, *I*^2^ = 0%) all showed significant improvement. Sensitivity analysis (pre- and post-intervention correlation coefficients *r*=0.3, 0.5, 0.7, 0.9) confirmed that BSFS and Wexner results were robust; GIQLI and CTT results are presented in tables due to the absence of forest plot data. Sensitivity analysis (*r* = 0.3–0.9) confirmed that BSFS, Wexner, GIQLI, and CTT results were robust; PAC-QOL and PAC-SYM were sensitive to correlation coefficients (Supplementary Table S5). Egger/Begg tests did not detect significant publication bias (*P* > 0.05) (Supplementary Table S6).

### 3.6 Cohort study outcomes

In four cohort studies (*n* = 887), meta-analysis was not conducted due to heterogeneity in study design and control groups (Supplementary Table S7). Lin (2020) showed that fresh and frozen FMT preparations had comparable efficacy (Tian et al., 2015). Tian et al. (2016a) showed that naso-intestinal tube administration had the best efficacy but lower compliance (70.0% vs. 85.0%), while oral capsules were easy to use and had fewer adverse events (Lin, 2020). Yang et al. (2018) showed that FMT combined with biofeedback was superior to biofeedback alone during follow-up of 1–12 months (Tian et al., 2016a). Zhang et al. (2021) showed that staged FMT (two courses, 1 month apart) had better efficacy at 12 weeks compared to a single FMT (Yang et al., 2021).

### 3.7 Safety

Five RCTs reported treatment-related adverse events. None of the studies reported serious adverse events (SAEs). The most common adverse events in the FMT group were bloating, diarrhea, nausea, and increased flatulence, all of which resolved spontaneously; in the control group, these included abdominal pain, diarrhea, and dry mouth. The overall incidence of adverse events was comparable between the two groups during the follow-up periods (range: post-treatment to 24 weeks), suggesting that FMT does not increase additional short-term risk. No studies reported safety outcomes beyond 24 weeks, precluding assessment of long-term adverse effects (for details, see Supplementary Table S8).

## 4 Discussion

This study, through an innovative “three-module” analytical framework, systematically integrates 12 RCTs, 16 single-arm studies, and 4 cohort studies (a total of 32 studies, *n* = 1,710 patients) for the first time, comprehensively evaluating the efficacy and safety of FMT in treating adult chronic constipation. The main findings are as follows: (1) Evidence from RCTs shows that FMT significantly improves the overall clinical effective rate (RR = 1.36, 95% CI: 1.21–1.52) and markedly improves KESS, Wexner scores, serum SP, and anxiety and depression scores; (2)While lacking control groups, single-arm studies provide exploratory complementary evidence suggesting FMT may improve constipation symptoms, with a combined remission rate of 51.7% and an improvement rate of 67.0%, and within-group improvements in BSFS, Wexner, GIQLI, and CTT. These within-group improvements cannot distinguish true FMT effects from placebo responses or natural disease fluctuations, and thus serve as hypothesis-generating rather than confirmatory evidence. (3) Cohort studies suggest that repeated FMT courses are superior to single treatments, FMT combined with biofeedback is superior to biofeedback alone, and the naso-intestinal route achieves the best efficacy while oral capsules have better adherence; (4) During follow-up periods of up to 24 weeks, FMT was well-tolerated with no serious adverse events reported, and the most common mild to moderate gastrointestinal symptoms resolved spontaneously. However, asserting definitive long-term safety profiles based entirely on short-term observational data overreaches the evidence; long-term adverse effects, including potential microbiota-related risks (e.g., metabolic disturbances, opportunistic infections), remain unknown. These short-term safety findings provide preliminary evidence, but long-term surveillance is required before FMT can be considered for routine clinical use.

So far, Wang et al. (2025) and Fang et al. (2021) have respectively published meta-analyses specifically targeting FMT treatment for chronic constipation. Wang et al. included 9 studies (*n* = 245), mostly single-arm (8/9), with only one RCT, did not cover Chinese literature, and did not perform paired before-and-after analyses. Fang et al. (2021) although including 5 RCTs (*n* = 409), focused only on the combined treatment strategy of FMT plus laxatives, did not systematically evaluate the efficacy of FMT monotherapy, and did not include data from single-arm studies or cohort studies (Fang et al., 2021). This study significantly enhanced the robustness and generalizability of the evidence by expanding the search scope (Chinese and English databases), introducing a ‘three-module' framework, and including more diverse study designs (12 RCTs, 13 single-arm studies, 5 cohort studies). Notably, the remission rate (50.7%) and BSFS improvement (MD = 1.32) reported by Wang et al.were highly consistent with the results of this study, suggesting that the efficacy of FMT is reproducible (Wang et al., 2025). However, this study, for the first time using an RCT design, confirmed that the relative efficacy of FMT is superior to conventional treatment (laxatives/lactulose), which is a key clinical question that single-arm studies cannot answer.

The biological mechanisms by which FMT improves constipation remain speculative and require mechanistic validation. Preliminary observations from the four microbiome analysis studies (all using 16S rRNA sequencing without metagenomic or metabolomic integration) suggest that after FMT, beneficial bacteria (Bifidobacterium, Prevotella, Fusicatenibacter) may be enriched, while pro-inflammatory bacteria (Enterobacteriaceae) may be suppressed (Zhang et al., 2021; Tian et al., 2016b; Tian, 2020; Zhang, 2017). However, these proposed pathways are based on taxonomic correlations rather than functional metagenomic or metabolomic evidence, and should be interpreted as hypothesis-generating. Bifidobacterium could potentially contribute to the gut-brain axis signaling by producing acetate and GABA, and butyrate-producing bacteria such as Fusicatenibacter might potentially influence the enteric nervous system and enhance colonic motility (Tian et al., 2020; Lanza et al., 2021). Secondly, in terms of gastrointestinal hormone regulation, RCTs showed that FMT significantly increased serum SP (SMD = 1.41, 95% CI: 1.01–1.81, *I*^2^ = 0%), which is consistent with the hypothesis that FMT may be associated with altered gut neurotransmitter balance (Lanza et al., 2021). However, this observation alone does not establish causal mechanisms and may be subject to placebo effects in open-label trials. Serum gastrointestinal hormones (MTL and VIP). Serum MTL and VIP results demonstrated extreme heterogeneity (*I*^2^ > 98%) with inconsistent effect directions across studies. We therefore explicitly caution against interpreting these findings as evidence for FMT-mediated neurohormonal modulation. The observed variability likely reflects substantial methodological differences: (1) assay heterogeneity—studies used different detection platforms (ELISA, chemiluminescence immunoassay, and radioimmunoassay) with varying sensitivities, specificities, and reference ranges; (2) pre-analytical factors—differences in sample collection timing (fasting vs. postprandial), storage temperature, centrifugation protocols, and freeze-thaw cycles; (3) physiological variability—MTL and VIP exhibit circadian fluctuations and are influenced by concurrent medications (e.g., prokinetics, laxatives) that were not standardized across trials; and (4) subtype-specific responses—STC and FC may differ in baseline enteric neuroendocrine profiles, yet no study stratified hormone analyses by subtype. Given these limitations, we recommend that MTL and VIP not be used as surrogate efficacy endpoints for FMT in chronic constipation until standardized collection, processing, and assay protocols are established. Thirdly, regarding improvement of psychological comorbidities, SAS and SDS scores were significantly reduced (SMDs of −0.81 and −1.13, respectively), supporting the significance of the “microbiota-gut-brain axis” interaction hypothesis in functional gastrointestinal disorders (El-Salhy et al., 2020). While this is consistent with the “microbiota-gut-brain axis” hypothesis, the observed improvements in subjective psychological outcomes in unblinded trials are particularly susceptible to placebo effects, and cannot be attributed to microbiota-mediated mechanisms without sham-controlled validation.

Sources of extreme heterogeneity in BSFS and PAC-QOL. The exceptionally high heterogeneity observed for BSFS (*I*^2^ = 96.4%) and PAC-QOL (*I*^2^ = 96.8%) warrants careful clinical interpretation beyond statistical modeling. Several factors likely contributed to this variability. First, constipation subtypes differed across studies: slow-transit constipation and functional constipation may exhibit divergent stool form responses to FMT due to distinct underlying microbiome profiles (Cammarota et al., 2017), yet only 5 of 16 studies explicitly reported subtype-stratified BSFS data, precluding reliable subgroup analysis. Second, donor characteristics varied considerably—fresh vs. frozen preparations, universal vs. related donors, and different stool processing protocols may differentially affect microbiota engraftment and thus stool consistency (Zhao and Yu, 2016), Third, administration routes (nasoenteric tube, gastroscopy, oral capsule, colonoscopy) introduced procedural heterogeneity that likely influenced microbial colonization patterns and downstream patient-reported outcomes (Kootte et al., 2017). Fourth, baseline severity varied widely: studies enrolling more severely constipated populations may demonstrate larger absolute BSFS improvements than those with milder disease, inflating between-study variance. Fifth, the timing of outcome assessment ranged from immediately post-treatment to 24 weeks; microbiota-mediated changes in stool form may be transient, and PAC-QOL, as a subjective patient-reported outcome, is particularly susceptible to placebo effects and recall bias in unblinded single-arm designs (Kao et al., 2017). Finally, dietary fiber intake and concurrent laxative use were poorly reported across studies, yet both directly influence BSFS and gut motility (Enck and Klosterhalfen, 2005; Eswaran et al., 2013). Future individual patient data (IPD) meta-analyses should formally test these moderators.

This study provides three key pieces of information for clinical decision-making of FMT: (1) Choice of administration route: Nasointestinal tube shows the best efficacy (74.2% improvement rate at 1 month), but the procedure is complex, with low compliance (70%) and multiple procedure-related adverse events (dyspnea, pharyngalgia); oral capsules are convenient, low-cost, highly compliant (85%), but slightly less effective (60%). For refractory constipation, the nasointestinal tube may be the primary choice; for patients requiring repeated treatment, oral capsules are more sustainable. (2) Combined treatment strategies: FMT combined with biofeedback continuously outperformed biofeedback alone in 1–12 month follow-ups (Tian, 2020), suggesting that FMT may enhance the long-term efficacy of pelvic floor rehabilitation through microbiota remodeling. RCTs of FMT combined with laxatives (PEG/lactulose) also showed synergistic effects, but the optimal combination regimen still needs exploration. (3) Treatment course design: phased FMT (repeat treatment intervals of 1 month) over 12 weeks is more effective than a single treatment, suggesting that gut microbiota colonization may require multiple reinforcement treatments, which is consistent with the ‘induction-maintenance' strategy of FMT in inflammatory bowel disease (Carabotti et al., 2015).

However, this study has the following limitations (Suares and Ford, 2011). Risk of bias and its impact on effect estimates: All RCTs were rated as high risk of bias due to the impossibility of blinding the FMT procedure; single-arm studies lack controls, making it impossible to distinguish FMT efficacy from placebo effects or natural disease fluctuations. The high risk of bias in all included RCTs has important implications for interpreting the observed efficacy. Lack of participant blinding in open-label FMT trials may inflate treatment effects for subjective outcomes (e.g., symptom scores, quality of life) by 15–30% based on empirical evidence from other medical fields. The absence of sham-controlled designs prevents distinction between true microbiota-mediated effects and placebo responses. Therefore, the adjusted RR of 1.21 may still overestimate the true efficacy of FMT, and the actual effect could be smaller or even null. The Harbord test suggests that there may be publication bias in the overall efficacy of RCTs (*P* = 0.003), potentially overestimating the true effect (Anorectal Branch of Chinese Medical Doctor Association, 2024). Heterogeneity and analytical limitations: Several primary outcomes exhibited exceptionally high statistical heterogeneity (BSFS: *I*^2^ = 96.4%; PAC-QOL: *I*^2^ = 96.8%; MTL: *I*^2^ = 98.3%; VIP: *I*^2^ = 99.1%). While random-effects models were used to account for between-study variance, reliance on these models alone without comprehensive subgroup exploration represents a significant analytical limitation. We conducted subgroup analyses for the primary outcome of overall clinical efficacy by administration route, treatment course, and constipation type (Figure 5, Supplementary Table S3); however, no significant subgroup interactions were identified (all *P_*interaction > 0.05). For outcomes with high heterogeneity, subgroup analyses were precluded by the small number of studies reporting these continuous outcomes (*n* = 2–5 per outcome), yielding insufficient statistical power for reliable stratification. Additionally, incomplete covariate reporting in original studies (e.g., constipation subtype was unspecified in 2 of 5 BSFS studies) further limited exploratory analyses. Although sensitivity analyses (leave-one-out) were conducted, these cannot substitute for systematic investigation of clinical and methodological sources of heterogeneity. Future individual patient data (IPD) meta-analyses or large-scale multicenter RCTs with standardized protocols are needed to address this gap (Bharucha and Wald, 2019). Geographic, dietary, and ethnic limitations. All 32 included studies were conducted in China, which significantly constrains global generalizability. Gut microbiota composition differs substantially between East Asian and Western populations due to distinct long-term dietary patterns: traditional Chinese diets are typically higher in fiber and complex carbohydrates (favoring Prevotella-dominant enterotypes), whereas Western diets are higher in fat and protein (favoring Bacteroides) (R Core Team, 2024; Arumugam et al., 2011). These enterotype differences may affect both donor microbiota composition and recipient colonization efficiency after FMT (Wu et al., 2011). Ethnic and genetic backgrounds also influence host immune responses to transplanted microbiota and baseline gut barrier integrity (Yatsunenko et al., 2012). Furthermore, diagnostic practices and constipation phenotyping may vary across healthcare systems; for example, the threshold for diagnosing STC based on colonic transit time may differ, and patient expectations toward FMT as a “natural” therapy may inflate placebo responses in Chinese cultural contexts. Finally, donor screening criteria and stool banking practices in China may not be directly transferable to other regulatory environments (e.g., FDA or EMA guidelines) (Goodrich et al., 2014). The complete absence of non-Chinese studies precludes any assessment of whether the observed FMT efficacy is reproducible in diverse ethnic, dietary, and regulatory contexts. Future multinational trials with standardized donor screening, outcome measures, and dietary stratification are essential to establish external validity. Additionally, the longest follow-up was 24 weeks, so long-term efficacy and persistence of gut microbiota colonization are unknown; the lack of RCTs with sham surgery controls prevents exclusion of strong placebo effects. (4) Mechanistic claims exceed evidence base: Although the manuscript discusses potential biological mechanisms, only four of 32 included studies (12.5%) performed microbiome analyses, all restricted to standard 16S rRNA sequencing without metagenomic, metabolomic, or functional validation. Consequently, the proposed mechanistic pathways (e.g., butyrate-mediated enteric nervous system activation, gut-brain axis restoration) are speculative and based on taxonomic correlations rather than causal evidence. Multi-omics integration and experimental validation in germ-free models are essential before these mechanisms can be considered established; (5) Follow-up duration and long-term safety: The maximum follow-up duration among included studies was 24 weeks, with most studies assessing outcomes at ≤ 12 weeks. This precludes evaluation of long-term efficacy durability, sustained microbiota colonization, and delayed adverse events (e.g., metabolic sequelae, infections, or malignancy risks associated with microbiota manipulation). All safety conclusions are therefore limited to the short-term observation period and should not be extrapolated to long-term FMT use. (6) Incomplete follow-up reporting: Follow-up duration was not reported in 58% of RCTs and 50% of single-arm studies, precluding stratified analyses by follow-up duration and systematic assessment of efficacy durability. Although leave-one-out sensitivity analyses demonstrated stable pooled estimates (Supplementary Table S4), the inability to evaluate long-term outcomes (beyond 24 weeks) reflects a broader reporting deficiency in the FMT for constipation literature. Future studies should adopt standardized follow-up protocols and explicitly report all time points.

Future studies should prioritize: large-sample, multicenter, double-blind RCTs with sham surgery controls; standardized donor screening and microbiota library construction; long-term follow-up (>1 year) to assess durability of efficacy and safety; multi-omics integration to identify predictive biomarkers of FMT response; exploration of optimal FMT dosage, administration route, and treatment regimen.

## 5 Conclusion

This study systematically evaluated the efficacy and safety of fecal microbiota transplantation (FMT) for the treatment of adult chronic constipation through an innovative ‘three-module' analysis framework. RCT evidence confirmed that FMT is superior to conventional treatment in improving overall clinical efficacy (RR = 1.21). Robust improvements were observed for Wexner score, KESS, serum SP, and psychological outcomes (SAS, SDS). However, due to extreme heterogeneity and inconsistent effect directions, no reliable conclusions can be drawn regarding FMT's effects on serum MTL or VIP. Single-arm studies, while lacking control groups, observed within-group improvements in symptoms and quality of life that require validation in controlled trials. Within the limited follow-up durations reported (≤24 weeks), FMT appeared to have a favorable short-term safety profile. Long-term safety data are lacking, and sustained monitoring is needed to exclude delayed adverse effects. Specifically, the pooled remission rate of single-arm studies was 51.7% (95% CI: 43.2%−60.2%), and the improvement rate was 67.0% (95% CI: 58.5%−74.8%); evidence from RCTs further confirmed that FMT was significantly superior to conventional treatment in terms of overall clinical efficacy (RR = 1.36, 95% CI: 1.21–1.52) (1). Cohort studies suggest that repeated FMT courses (Yang et al., 2021) and combined therapeutic strategies (such as FMT combined with biofeedback) (Tian, 2020) may enhance efficacy, and oral capsules have better compliance than nasointestinal tubes (Lin, 2020).

From a mechanistic perspective, limited and preliminary microbiome analyses from only four of 32 included studies (12.5%), all restricted to 16S rRNA sequencing without metagenomic or metabolomic integration, suggest that FMT may be associated with alterations in gut bacterial composition:beneficial bacteria may be enriched, including Bifidobacterium (phylum Actinobacteria) (Zhang et al., 2021), Prevotella and Paraprevotella (phylum Bacteroidetes) (Xie et al., 2021), as well as butyrate-producing Fusicatenibacter and Megamonas (phylum Firmicutes) (Zhang, 2017); meanwhile, pro-inflammatory Enterobacteriaceae (phylum Proteobacteria) may be suppressed (Tian, 2020). However, these taxonomic observations cannot establish causal mechanistic pathways. The proposed roles of microbially derived metabolites (e.g., butyrate, acetate) in gut-brain axis signaling remain hypothetical— butyrate could potentially influence the enteric nervous system and enhance colonic motility (Tian et al., 2020), while acetate regulates visceral sensitivity via vagal nerve signaling (Lanza et al., 2021). These proposed pathways require experimental validation before they can be considered established mechanisms.

Clinical practice recommendations based on current evidence: Given the very low to moderate certainty of evidence and high risk of bias in all included studies, we do not recommend FMT as a standard treatment for chronic constipation outside of clinical trials. Patients interested in FMT should be informed that: (1) the true efficacy remains uncertain due to methodological limitations of existing studies; (2) benefits may be smaller than reported; (3) long-term safety data are lacking. FMT should be offered only within well-designed clinical trials or regulated compassionate-use programs with rigorous informed consent.

However, clinical outcomes show significant heterogeneity (most indicators *I*^2^ > 70%), arising from differences in donor selection criteria, routes of administration, and follow-up durations (Wang et al., 2025). Most studies were open-label designs and lacked sham-controlled RCTs, making it impossible to rule out the placebo effect and natural disease fluctuations (Pokusaeva et al., 2017). In addition, all included studies were conducted in China, limiting generalizability (El-Salhy et al., 2020).

In summary, (1)despite a statistically significant pooled effect (adjusted RR = 1.21, 95% CI: 1.10–1.33), the certainty of evidence is very low to moderate due to high risk of bias in all included RCTs, substantial heterogeneity, and suspected publication bias. FMT shows potential as a therapeutic candidate for chronic constipation, but current evidence is insufficient to support definitive clinical recommendations. These findings should be considered hypothesis-generating and require validation in large-scale, double-blind, sham-controlled RCTs before routine clinical implementation; (2) standardized donor selection and stool bank construction based on metagenomic analyses (Carabotti et al., 2015); (3) long-term follow-up (>24 weeks) multi-omics integrative analyses to distinguish durable microbiota colonization from transient ecological shifts (Paramsothy et al., 2017); (4) mechanistic validation of microbiota-derived metabolites in germ-free animal models (Fehily et al., 2021). Only through these coordinated efforts can FMT be transformed from empirical practice into targeted, ecosystem-level precision therapy that addresses refractory constipation through sustained microbiota-host interactions rather than merely symptomatic relief.

## Data Availability

The original contributions presented in the study are included in the article/Supplementary material, further inquiries can be directed to the corresponding authors.
